# Functional Characterization of the First *Bona Fide* Phytoene Synthase in Red Algae from *Pyropia yezoensis*

**DOI:** 10.3390/md22060257

**Published:** 2024-05-31

**Authors:** Cheng-Ling Li, Jia-Qiu Pu, Wei Zhou, Chuan-Ming Hu, Yin-Yin Deng, Ying-Ying Sun, Li-En Yang

**Affiliations:** 1 School of Marine Science and Fisheries, Jiangsu Ocean University, Lianyungang 222005, China; 2 Jiangsu Marine Fisheries Research Institute, Nantong 226007, China; 3 College of Marine Sciences, Shanghai Ocean University, Shanghai 201306, China

**Keywords:** carotenoid biosynthesis, phytoene synthase, functional characterization, red algae

## Abstract

The formation of phytoene by condensing two geranylgeranyl diphosphate molecules catalyzed by phytoene synthase (PSY) is the first committed and rate-limiting step in carotenoid biosynthesis, which has been extensively investigated in bacteria, land plants and microalgae. However, this step in macroalgae remains unknown. In the present study, a gene encoding putative phytoene synthase was cloned from the economic red alga *Pyropia yezoensis*—a species that has long been used in food and pharmaceuticals. The conservative motifs/domains and the tertiary structure predicted using bioinformatic tools suggested that the cloned *PyPSY* should encode a phytoene synthase; this was empirically confirmed by pigment complementation in *E. coli*. This phytoene synthase was encoded by a single copy gene, whose expression was presumably regulated by many factors. The phylogenetic relationship of PSYs from different organisms suggested that red algae are probably the progeny of primary endosymbiosis and plastid donors of secondary endosymbiosis.

## 1. Introduction

The biosynthesis of carotenoids starts with condensing two geranylgeranyl diphosphate (GGPP) molecules by phytoene synthase (PSY) to form phytoene, the rate-limiting step of the pathway [1]. Due to the crucial role of PSY in controlling the flux toward carotenoids, it is regulated by complex factors and mechanisms [2]. The overexpression or knockout of the *PSY* gene significantly enhanced or decreased the accumulation of downstream carotenoids, respectively, indicating solid transcription regulation [3,4,5]. The post-transcription regulations of *PSY* by the Orange (OR) protein or STAY-GREEN protein have also been reported recently [6,7]. 

The first *PSY* gene from land plants was characterized in *Solanum lycopersicum* [8,9]. To date, these genes have been characterized in many organisms, such as *Arabidopsis thaliana*, *Oryza sativa*, *Triticum aestivum*, etc. In some land plants, PSYs are encoded by a small gene family and have different functions [10]. The sub-functions of PSY enable the accumulation of carotenoids in non-photosynthetic tissues responding to environmental stresses and provide various regulatory mechanisms for carotenoid biosynthesis [2]. For example, in *Solanum lycopersicum*, there are three tissue-specific expression *PSY* genes. *PSY1* is responsible for carotenoid biosynthesis in fruits, while, under stress, *PSY2* functions in leaves and *PSY3* in roots [11]. However, with some exceptions, PSY is encoded by a single copy gene in most algae [12].

An analysis of the genes involved in carotenoid biosynthesis indicated that the carotenoid pathway originated from prokaryote, a probable common ancestor of archaea and bacteria that co-evolved with photosynthesis and transferred from endosymbiont cyanobacteria to the plastid in host plants during evolution [13]. This inference was supported by the evidence that the genes involved in the C40 biosynthetic pathway are conservative in archaea and bacteria, indicating that there was a common carotenogenic progenitor. The active site structure of extant PSYs resembles those of their progenitors, further proving the prokaryote origin of the carotenogenic genes [14]. The sub-functionalization of PSYs in land plants might be a result of gene duplication and subsequent loss from a common progenitor [10]. 

*Pyropia yezoensis* (formerly *Porphyra yezoensis*, and *Neopyropia yezoensis* [15]) classified in the new genus by Sutherland et al. (2011) [16]), an economic and edible red alga, is mainly cultivated and used for food in Asian countries, such as China, Japan and South Korea, producing approximately USD 2.19 billion annually. Nonetheless, studies of the carotenogenic gene in red algae have progressed slowly, with geranylgeranyl diphosphate synthase (GGPPS), lycopene cyclases (LCYs) and carotene hydroxylases (CHYs) being characterized from *Bangia fuscopurpurea*, *N. yezoensis* or *Porphyra umbilicalis*, respectively, in recent decades [17,18,19,20,21]. However, as the rate-limiting step, the number of copies of the genes encoding PSY and the nature of how they evolved and are regulated in red algae remain unknown. The present study cloned and functionally characterized the *PSY* in red algae for the first time. The probable regulatory mechanisms and evolution events are also discussed. 

## 2. Results

### 2.1. Cloning of PyPSY

**Figure 1:**
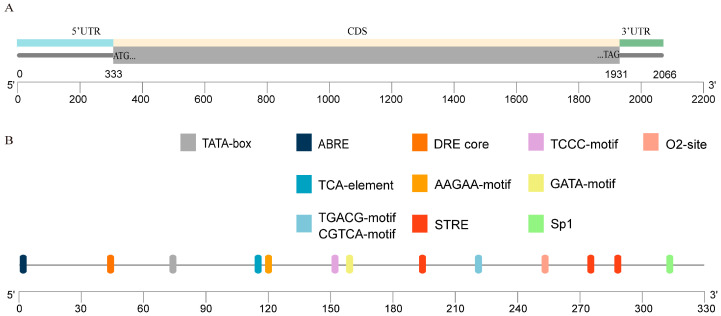
mRNA structure of *PyPSY* and transcript regulatory factors upstream of the gene. (**A**) Full-length mRNA obtained using RACE; (**B**) Transcript regulatory factors upstream of the gene.

The contigs py02040.t1 and contig_24954 were obtained from the *N. yezoensis* v1 CDS and CLC genome database, respectively, by blasting, using the *PSY* coding sequence of *Arabidopsis thaliana* (AT5G17230) as a query [6]. A hit, CM020618.1, was also found in NCBI. An analysis of these three contigs showed that they contain an identical ORF of 1599 bp. Primer pairs were designed according to the putative open reading frame (ORF) to amplify the gene from mRNA. After sequencing, the amplicon sequences had a high similarity with the contigs according to three base pair variations: C98T, G417A and T871G. The primer pair was also used to amplify the gene from genomic DNA from the start to the stop codon. The genome amplicon sequence was the same as that of ORF from cDNA, indicating that the gene was intron-less. Primer pairs were designed according to the ORF sequence obtained to amplify the full-length mRNA of the putative *PSY* gene from *N. yezoensis*. After sequencing and combining the amplicons, a 2066 bp of full-length mRNA encoding putative *PyPSY* was obtained, composed of 332 bp 5′-UTR, 1599 bp ORF and 135 bp 3′-UTR (Figure 1A). There were three variations in the 5′-UTR region and no variations in the 3′-UTR region compared with contig_24954. The obtained sequence was deposited in NCBI under accession number PP789597.

Using genome walking, a 259 bp region upstream of the start codon was obtained. This upstream region was consistent with that obtained from mRNA using RACE. Unfortunately, the upstream was no longer than the 5′-UTR of mRNA. The upstream sequence to the start codon ATG was analyzed for potential regulatory factors (Figure 1B). Four light-responsive cis-elements—TCCC-motif, GATA-motif, Sp1 and TATA-box—were identified. Three cis-acting elements responding to plant hormone abscisic acid (ABA), salicylic acid (SA), zein and methyl jasmonic acid (MeJA) were identified as ABRE, TCA and TGACG, respectively. An O2 site involved in zein regulation was also detected. Two cis-elements responsive to abiotic environmental factors were also discovered. The dehydration-responsive element (DRE) core sequence (GCCGAC) responds to desiccation, low temperatures and hypersaline stresses. The stress response element (STRE) is a cis-element responsive to desiccation, hypersalinity and heat shock.

### 2.2. Analysis of PyPSY Protein

The ORF of *PyPSY* encodes a protein composed of 532 amino acids, with an estimated molecular weight of 57.40 kDa and a pI of 5.72. The instability index was 49.9, suggesting that PyPSY is an unstable protein. The aliphatic index was 85.06, and the average hydrophilicity value was −0.125, indicating that PyPSY is moderately hydrophilic. The subcellular location of PyPSY was predicted to be in the plastid using WoLF PSORT; however, no signal peptide was detected by SignalP v6.0. The subcellular location predicted by DeepLoc was plastid, with a probability of 0.9776. According to the prediction by TargetP, the cleavage site of the signal peptide was between 60 and 64 amino acids. There was no transmembrane domain in PyPSY, which was predicted using TMHMM.

**Figure 2:**
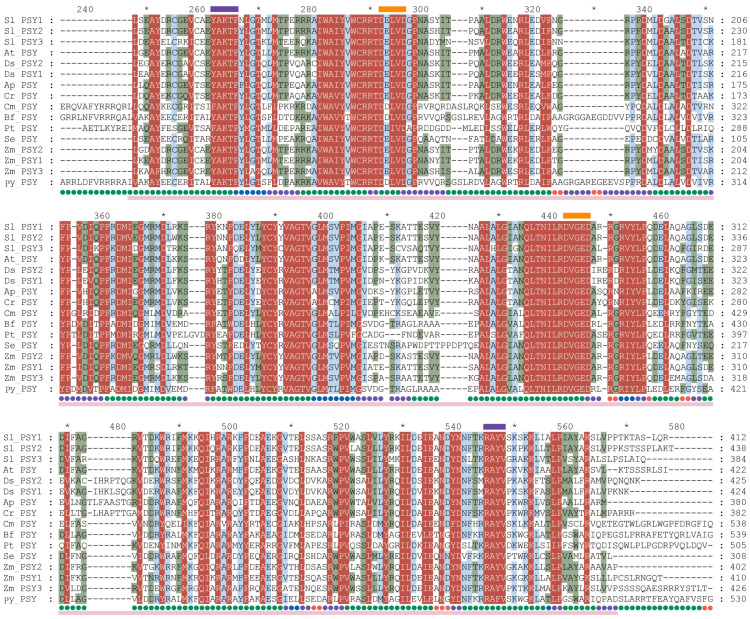
Conserved regions of PSY protein homologs from different organisms. The blue frame indicates two magnesium binding sites; and the orange frame indicates two aspartic-acid-rich motifs. Green dots indicate residues composed of α-helix in their secondary structure. Abbreviations of species names are as follows: Sl for *Solanum lycopersicum*, At for *Arabidopsis thaliana*, Ds for *Dunaliella salina*, Ap for *Auxenochlorella protothecoides*, Cr for *Chlamydomonas reinhardtii*, Cm for *Cyanidioschyzon merolae*, Bf for *Bangia fuscopurpurea*, Pt for *Phaeodactylum tricornutum*, Se for *Synechococcus elongatus*, Zm for *Zea mays* and Py for *Pyropia yezoensis*.

Domain analyses using CD Search and InterPro showed that all PSYs belong to the isoprenoid synthase superfamily and share a conserved prenyltransferase domain. A multi-alignment and motif analysis of the deduced PSY protein sequences from different organisms demonstrated that the active site residues, two aspartic-acid-rich motifs (DXXXD) and the magnesium binding site were highly conserved (Figure 2). One of the three variations in the gene was a synonymous mutation, and the other two resulted in two amino acid substitutions: Val33Ala and Ala291Ser. Both amino acid variations were located in the non-conserved region. In red algae, the second amino acid, Glu, of the first aspartic-acid-rich motif (FARM) was replaced by aspartic acid, making the motif DDXXD. The highest similarity occurred between the PyPSY and the putative PSY from *Porphyra umbilicalis* (PuPSY, 79.43%), followed by that from *Bangia fuscopurpurea* (BfPSY, 77.88%), both of which belong to the same family, Bangiaceae. The similarity between the PyPSY and the functional characterized AtPSY from *Arabidopsis thaliana* was 48.00%.

**Figure 3:**
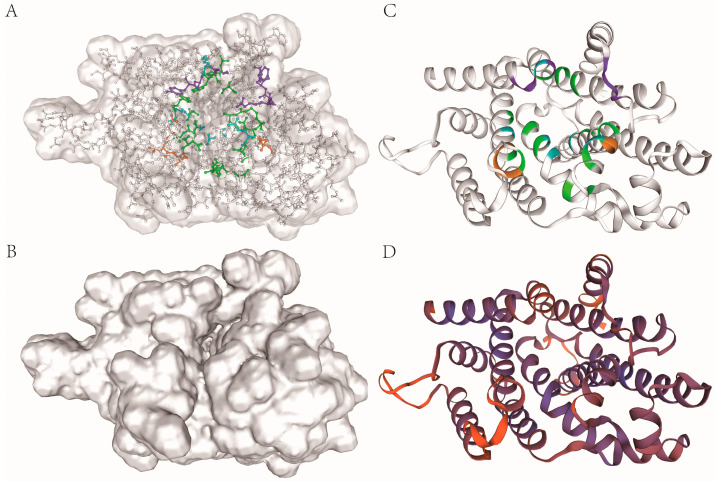
Tertiary structure of PyPSY. (**A**) The tertiary structure of PyPSY shows the active cavity and active site amino acid residues surrounding it, with green indicating catalytic residues, purple indicating active site lid residues, orange indicating DDXXD motifs and cyan indicating substrate-binding pockets; (**B**) the active cavity; (**C**) ribbon model of PyPSY with the same perspective and active amino acid residues (colors the same as in (**A**)); (**D**) shows the reliability of the tertiary structure of PyPSY, with purple representing more reliability.

To further predict the PyPSY function, its tertiary structure was constructed using homology modeling. The tertiary structure of PSY from *A. thaliana* was available when searching against the AlphaFold database using SWISS-MODEL; this was used as the template for homology modeling. The model showed that PyPSY probably functions as a monomer with a GQME value of 0.36 (Figure 3A). An active cavity surrounded by catalytic amino acid residues can dock the substrate GGPP by binding Mg^2+^ (Figure 3B). The cavity surrounded by active amino acids can also be seen in the ribbon model (Figure 3C). These results indicate that the putative PSY cloned from *N. yezoensis* might be a functional phytoene synthase. The reliability of the constructed model was tested, and different colors indicate the reliability of the constructed tertiary PyPSY structure (Figure 3D). The red color represents the unreliable folding of amino acid residues in the loops. The Ramachandran plot shows how reasonably folded amino acid residues accounted for more than 90%.

### 2.3. Functional Characterization of PyPSY

**Figure 4:**
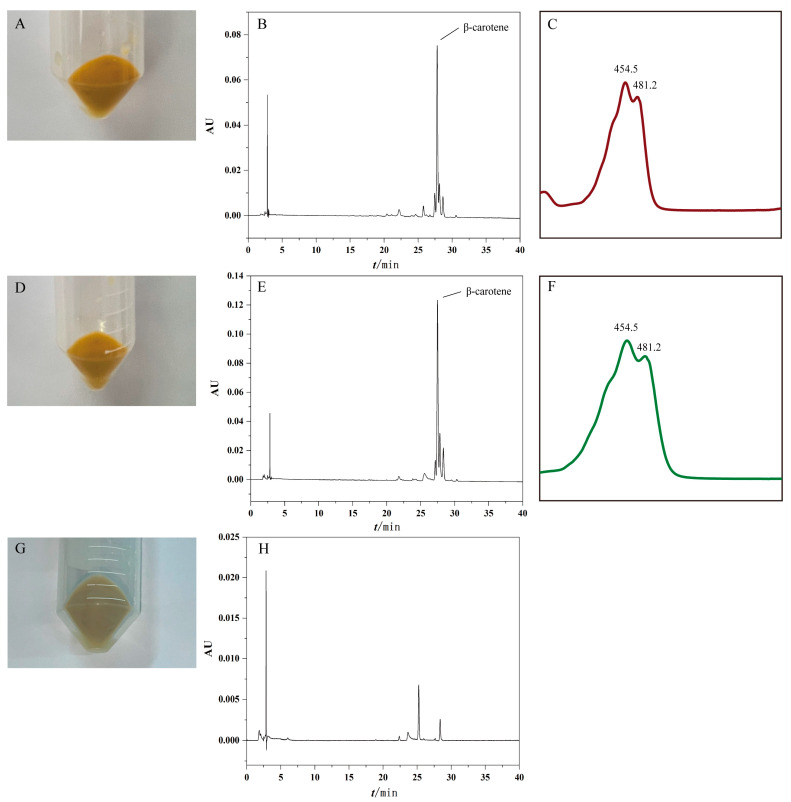
Functional characterization of PyPSY. The yellow color (**A**), the chromatograph (**B**) and absorbance spectra (**C**) of β-carotene accumulated in *E. coli* cells harboring pMAL-AaPSY and pAC-85b; the yellow color (**D**), the chromatograph (**E**) and absorbance spectra (**F**) of β-carotene accumulated in *E. coli* cells harboring pMAL-PyPSY and pAC-85b; the white color (**G**) and the chromatograph (**H**) of unknown components accumulated in *E. coli* cells harboring pMAL-c5x and pAC-85b.

Since PyPSY is an unstable protein, a pigment complementation platform in *Escherichia coli* was employed to characterize its function. To accomplish this, the ORF of *PyPSY* was cloned into the expression vector pMAL-c5x and designated it as pMAL-PyPSY. The target gene harboring in the vector was verified by sequencing. When co-transferred with pAC-85b, which harbors genes encoding geranylgeranyl diphosphate synthase (*crtE*), phytoene desaturase (*crtI*) and lycopene β-cyclase (*crtY*) from *Pantoea agglomerans* Eho 10, the *E. coli* colonies should turn yellow if PyPSY can function as a phytoene synthase. The results showed that *E. coli* colonies harboring pMAL-PyPSY and pAC-85b turned yellow, as did the positive control harboring pMAL-AaPSY and pAC-85b (Figure 4A,D). In contrast, the negative control remained white (Figure 4G).

The pigments in each group were extracted and analyzed using HPLC. Chromatographic peaks were detected in both the sample and positive control, which were determined to be β-carotene based on the retention time and absorbance spectra (Figure 4B,C,E,F). Similarly to the positive control, the amount of β-carotene accumulated in *E. coli* cells harboring pMAL-PyPSY and pAC-85b indicated that PyPSY could synthesize phytoene using GGPP as a substrate provided by crtE. The phytoene was provided for crtI and crtY to synthesize β-carotene (Figure 4B,E). On the contrary, no β-carotene was detected in negative control cells harboring the empty vectors pMAL-c5x and pAC-85b (Figure 4H).

### 2.4. Evolution of PSY

**Figure 5:**
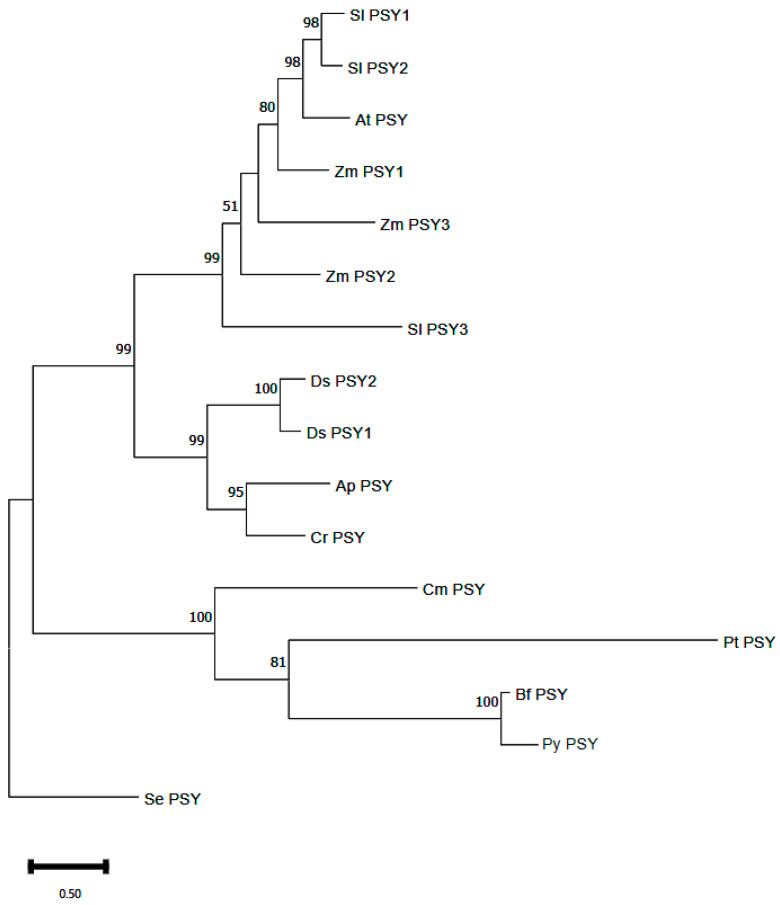
Phylogenetic relationships of PSYs from different organisms based on amino acid sequences. Abbreviations are as follows: Sl for *Solanum lycopersicum*, At for *Arabidopsis thaliana*, Zm for *Zea mays*, Ds for *Dunaliella salina*, Ap for *Auxenochlorella protothecoides*, Cr for *Chlamydomonas reinhardtii*, Cm for *Cyanidioschyzon merolae*, Pt for *Phaeodactylum tricornutum*, Bf for *Bangia fuscopurpurea*, Py for *Pyropia yezoensis* and Se for *Synechococcus elongatus*.

A phylogenetic tree reconstructed with the protein sequences of PSY from different organisms showed that PSY from Bangiaceae species clustered together first, forming a sister clade with the diatom *Phaeodactylum tricornutum*. The unicellular red alga *Cyanidioschyzon merolae* was located at their base. The green algae and higher plants were clustered in a sister clade of red algae and heterokonts. In this clade, green algae could be clearly separated from higher plants, but different copies of PSY in the same organism were not clustered in the same clade (Figure 5). 

## 3. Discussion

There is a growing interest in developing new plant varieties or cultivars with higher carotenoid contents. As an economically important red alga in food, *N. yezoensis* is a good candidate for metabolic carotenoid engineering. The biosynthesis of phytoene catalyzed by phytoene synthase is a rate-limiting step responsible for the terpenoid flux toward carotenoids [6]. Given its crucial role in controlling the size of the carotenoid pool, this study determines how many phytoene synthases are present in *N. yezoensis*, as well as their functions.

Because of the conservation of carotenoid biosynthesis, *PSY* has been extensively investigated via homology searching and pigment complementary characterization, especially in crops, fruits and microalgae [6,10]. It has been predicted that carotenoid biosynthetic genes are present in many algae [12]; however, no phytoene synthase has been functionally characterized. Although carotenoid biosynthesis is conservative among most organisms, algae present some differences. For example, scarcely any carotene hydroxylase CYP97B activity is seen in land plants such as *A. thaliana* [22], whereas only functional carotene hydroxylase activities occur in *P. umbilicalis* [17]. Therefore, it is necessary to empirically confirm this pathway in algae. The present study cloned a putative gene encoding phytoene synthase from the economic red alga *N. yezoensis*. The deduced protein was predicted to be unstable and localized in the plastid, consistent with those in land plants [2]. Sequence alignment and homology modeling also revealed a strong signal that this cloned gene from *N. yezoensis* encodes a putative phytoene synthase, which was empirically confirmed by pigment complementation in *E. coli*. This is the first bona fide PSY conferring phytoene synthase activity characterized in red algae. 

In some land plants, such as *Solanum lycopersicum* (tomato) [23], *Oryza sativa* (rice) [24], *Zea mays* (maize) [25], etc., PSYs are encoded by a small gene family composed of two or more orthologs, which exhibit organ or environmental stimuli specificity [26,27]. In *A. thaliana*, a genome-wide survey indicated that PSY is encoded by a single gene copy [28]. The alternative splicing of *PSY* in *A. thaliana* might encode multiple PSY isoenzymes to respond to environmental cues [29]. Unlike land plants, searches against transcriptome data revealed that most algae possess a single gene copy [12]. *Dunaliella salina* is one of the exceptions, in which two genes encoding PSY were predicted. PSY1 in *Dunaliella salina* CCAP 19/19 showed high activity, while PSY2 barely showed any [30]. Whether this is the situation in other algae with more than one gene encoding PSY remains unknown. In the present study, the searches against all available databases detected a single copy gene of *PSY* in *N. yezoensis*. Previous prediction using transcriptome data from *P. umbilicalis*, a species in the same family Bangiaceae as *N. yezoensis*, also detected a single copy gene for PSY [17]. Further research is needed to understand how a single copy PSY is regulated to control carotenogenesis in *N. yezoensis* that survive in their ever-changing intertidal environment. Furthermore, all enzymes involved in carotenoid biosynthesis are encoded by single copy genes in *P. umbilicalis*. Although Wang et al. (2018) reported that *N. yezoensis* encodes two phytoene desaturases [12], only one could be identified (data not shown). The simple suite of carotenoid biosynthesis in Bangiaceae species might be related to their simple morphogenesis without any tissue/organ differentiation, except for rhizoid and reproductive cells.

A considerable number of studies revealed that *PSY* regulation is a complex and multi-faceted regulatory machinery and network composed of transcriptional, post-transcriptional and post-translational tiers [2]. It is known that transcriptional regulation is central to controlling PSY activity for carotenogenesis. Various abiotic or biotic factors and signals, including light, salt, drought, temperature, development and hormones, have been identified as regulating *PSY* expression. *N. yezoensis* is an intertidal alga that suffers from the daily, rhythmic, tidal variations in light intensity, temperature and aridity. In the present study, thirteen cis-acting elements responding to light, phytohormones, drought, low temperature, heat shock and hydrosalinity were predicted in the short upstream region (332 bp) of *PyPSY*. Investigations into more extended upstream regions would undoubtedly reveal more cis-acting elements [31], which, to some extent, might compensate for the single copy gene encoding PSY. Studies on carotenoid biosynthesis regulation in red algae are rare. Regarding *Bangia fuscopurpurea*, another Bangiaceae species living in the intertidal niche, Deng et al. (2020) demonstrated that its PSY expression was regulated by circadian lighting, light intensity and aridity [21]. A previous study confirmed that PyPSY expression could be regulated by desiccation to control the pool size of carotenoids functioning as antioxidants [32]. It is reasonable to speculate that carotenoids controlled by *PSY*, responding to various biotic or abiotic clues, might play a critical role in red algae survival in the ever-changing intertidal environment [33]. 

Abscisic acid (ABA) is one of the most essential phytohormones in land plants, and it responds to environmental signals, such as drought and low temperature. The biosynthetic pathway of ABA was elucidated in land plants as a derivative of carotenoids [34]. Although the content of ABA in algae is lower than in land plants, it has been detected in *N. yezoensis* [35]. Nonetheless, the predicted biosynthetic pathway of ABA in *N. yezoensis* was ambiguous, with some enzymes absent (unpublished results). ABA can regulate the biosynthesis of its precursors by interacting with cis-acting elements that control the *PSY* expression [34]. It would be interesting to know whether the predicted ARE in the upstream region of *PyPSY* plays such a role. DRE is a cis-element responsive to drought, heat shock and hypersalinity stresses through its interaction with the transcription factor DREB in land plants [36]. In tobacco, by binding to DRE in the promoter of *PSY*, NtDREB-1BL1 can regulate carotenoid biosynthesis. The overexpression of *NtDREB-1BL1* upregulates the expression of *PSY* and enhances the drought tolerance of the transgenic line [37]. Previous studies demonstrated that carotenoids participate in aridity adaptations in *N. yezoensis* [32]; the DRE present in *PyPSY* might function similarly. The three STREs predicted in *PyPSY* signal that it responds to various stresses, emphasizing the regulation of PSY-controlled carotenogenesis in red algae.

PSYs in all carotenogenesis organisms phylogenetically belong to two super-classes [30]. In the present study, the single copy bona fide PSY, characterized and cloned from *N. yezoensis*, belonged to Class II. Ancient gene duplication occurred before establishing red and green lineages, and most of the red and green members retained one of them [2]. Gene duplications occurred more frequently in land plants, especially in Poaceae, but not in most algae [25]. The basal part of the phylogenetic tree is consistent with that of carotene hydroxylases and lycopene cyclases [17,20]. This consistency indicates that the carotenoid biosynthetic pathway in Archaeplastida might originate from prokaryotic cyanobacteria. After the splitting of green and red lineages, some unicellular red algae may be plastid donors in heterokonts, such as brown algae and diatoms. Subsequent gene duplication and sub-functionalization occurred in some of the lineages.

## 4. Materials and Methods

The haploid foliose of *Pyropia yezoensis* was collected from the offshore cultivation farm in Nantong, Jiangsu Province. The samples were transported to the laboratory at a low temperature and rinsed with boiled seawater; epiphytes were removed, and the samples were stored in a −80 °C refrigerator for further use. 

To obtain the putative gene encoding phytoene synthase in *N. yezoensis*, the functionally characterized PSY gene coding sequence (CDS) of *Arabidopsis thaliana* was used as a query to blast the genome database of *N. yezoensis* (http://porphyra.rutgers.edu/ (accessed on 2 June 2022)). All three databases were blasted using default parameters, including CLS genome scaffolds, v1 CDS and v1 genome databases. The obtained contigs were aligned (http://multalin.toulouse.inra.fr/multalin/ (accessed on 3 June 2022)) and analyzed for their ORF using online tools (http://www.bio-soft.net/sms/ (accessed on 3 June 2022)).

Genomic DNA and total RNA were extracted from the foliose of *N. yezoensis* using the improved methods of Yang et al. (2013) [38]. According to the manufacturer’s instructions, the first-strand cDNA was synthesized using the PrimeScript™ 1st Strand cDNA Synthesis Kit (Takara, China). To determine the gene structure of this putative phytoene synthase, the primer pair PyPSY-HF/PyPSY-ER was designed to amplify the ORF from the genomic DNA and cDNA of *N. yezoensis*, respectively, to determine whether the gene contained any intron in its coding region. The PCR was conducted on a thermocycler (Biometra, Germany) using an annealing temperature of 55 °C. All primers used in the present study are listed in Table 1. RACE-ready cDNA was synthesized using the SMARTer RACE 5′/3′ Kit (Takara), following the manufacturer’s instructions. A nested PCR was conducted to obtain the full-length mRNA of *PyPSY* using primer pairs PyPSY-SF/PyPSY-SR and PyPSY-NSF/PyPSY-NSR. To obtain the upstream sequence of PyPSY, genome walking was conducted using a Universal Genome Walker 2.0 kit (Takara). The nested PCR primers PyPSY-GR and PyPSY-NGR were used in the first- and second-round PCR, respectively. All amplicons were sequenced directly and combined with Vector NTI (10.0). The upstream flanking sequence was analyzed for cis-acting elements using the online tool PlantCARE (https://bioinformatics.psb.ugent.be/webtools/plantcare/html/ (accessed on 13 November 2022)).

The protein sequence of the putative phytoene synthase in *N. yezoensis* was deduced from the obtained ORF sequence using SnapGene software (www.snapgene.com (accessed on 15 November 2022)). To determine whether the PSY was a putative functional phytoene synthase, functionally characterized PSY proteins from red algae, green algae and land plants were searched. Three PSY copies of *Solanum lycopersicum* and *Zea mays*, two copies of *Dunaliella salina* and one copy of *Chlamydomonas reinhardtii*, as well as *Phaeodactylum tricornutum*, were included in the multi-alignment. Although their function was not characterized, PSYs from the red algae *Bangia fuscopurpurea* and *Cyanidioschyzon merolae* were also included in the analysis. The protein sequences were aligned using the ClustalW algorithm incorporated into MEGA v11.0. The conserved domains were searched using online tools NCBI-CD Search (https://www.ncbi.nlm.nih.gov/Structure/bwrpsb/bwrpsb.cg (accessed on 20 November 2022)) and InterPro (https://www.ebi.ac.uk/interpro/ (accessed on 20 November 2022)), and the conserved motifs were analyzed using MEME (https://meme-suite.org/meme/ (accessed on 21 Novermber 2022)). To determine the stability of PyPSY, the instability index, aliphatic index and the average hydrophilicity value were calculated using the online tool ProtParam (https://www.expasy.org/resources/protparam (accessed on 21 November 2022)). 

The biosynthesis of carotenoids in land plants was carried out in the plastid, and most of the carotenogenesis enzymes were membrane proteins. To determine the subcellular location of red algal PSY, the subcellular locations of PyPSY were predicted using WoLF PSORT (https://wolfpsort.hgc.jp/ (accessed on 24 November 2022)), SignalP (https://services.healthtech.dtu.dk/services/SignalP-6.0/ (accessed on 24 November 2022)) and DeepLoc (https://services.healthtech.dtu.dk/services/DeepLoc-2.0/ (accessed on 24 November 2022)), and the cleavage sites of signal peptides were predicted using TargetP (https://services.healthtech.dtu.dk/services/TargetP-2.0/ (accessed on 24 November 2022)). The transmembrane domain was predicted using TMHMM (https://services.healthtech.dtu.dk/services/TMHMM-2.0/ (accessed on 24 November 2022)).The functionally characterized PSY from *A. thaliana* [2,6], obtained from the alphafold database (https://www.alphafold.ebi.ac.uk/entry/P37271 (accessed on 17 December 2022)), was used as the template, and PyPSY homology modeling was conducted using the online tool SWISS-MODEL (https://swissmodel.expasy.org/ (accessed on 19 December 2022)). All parameters used in homology modeling were defaults. 

To characterize the function of PyPSY, the pigment complementary platform in *E. coli* was employed. The plasmid pAC-85b was purchased from Addgene. This plasmid was constructed by Cunningham et al. (2007) [39], harboring *crtE*, *CrtI* and *CrtY* from *Pantoea agglomerans* Eho 10. In the presence of phytoene synthase, *E. coli* cells harboring pAC-85b can accumulate β-carotene, turning the colonies yellow. An expression plasmid of *PyPSY* was constructed by cloning the ORF of *PyPSY* into pMAL-c5x using an In-Fusion^®^ Snap Assembly Kit (Takara). Primer pairs with homology sequences PyPSY-pF/PyPSY-pR were designed and used to amplify the ORF and cloned into the vector using homology recombination. The construct was co-transferred into *E. coli* cells with pAC-85b via the heat-shocking method used by Yang et al. (2014) [17]. The phytoene synthase gene from *Adonis aestivalis AaPSY* was synthesized into pMAL-c5x and designated as pMAL-AaPSY. The construct pMAL-AaPSY, co-transferred with pAC-85b, was used as the positive control, while the pMAL-c5x co-transferred with pAC-85b was the negative control. The transferred *E. coli* was screened on an Lysogeny Broth (LB) agarose medium with chloramphenicol and carbenicillin. The selected colonies were verified by colony PCR using the primer pair PyPSY-CF/PyPSY-CR and inoculated in LB medium with the same antibiotics at 250 rpm and 30 °C for a week. After inoculation, the *E. coli* cultures were centrifuged at 10,000× *g* for 5 min, and the pelleted cells were either stored at −80 °C or promptly used. The pigments were extracted and analyzed using previously reported methods [17]. The HPLC peak identities were determined via the retention time and ultraviolet/visible spectra recorded with the PDA detector (wavelengths from 295 to 600 nm) and the chromatography was monitored using 440 nm. 

Homologs encoding phytoene synthase from *A. thaliana* (dicot), *Solanum lycopersicum* (dicot), *Zea mays* (monocot), *Dunaliella salina* (green alga), *Chlamydomonas reinhardtii* (green alga), *Auxenochlorella protothecoides* (green alga), *Phaeodactylum tricornutum* (diatom), *Cyanidioschyzon merolae* (red alga), *Bangia fuscopurpurea* (red alga) and *Synechococcus elongatus* (cyanobacterium) were searched against GeneBank. These species represent the main evolutionary lineages involved in primary and secondary endosymbiosis. The amino acid sequences were aligned using ClustalX, and the best evolution model was tested using the MEGA-Model incorporated in MEGA. The maximum likelihood phylogenetic tree was reconstructed using MEGA, and bootstrap resampling (1000 replicates) was used to ensure the reliability of the tree. 

## Figures and Tables

**Table 1:** Primers used in the present study.

**Primer Name**	**Sequence**
For ORF	
PyPSY-HF	5′-ATGAGCCTCAATCCGCCGCCAATG-3′
PyPSY-ER	5′-CTACCCAGAACCAAACGACACAAACG-3′
For 5′-RACE	
PyPSY-SF	5′-GATTACGCCAAGCTCGCCGACGACTCGCAGGTGCATCGTTT-3′
PyPSY-NSF	5′-GATTACGCCAAGCTTGGCAAGGGAGCACCTGCAAAC-GCCAAGG-3′
For 3′-RACE	
PyPSY-SR	5′-GATTACGCCAAGCTCCTGGACATGTACGCCGG-CATCCTCGAGGT-3′
PyPSY-NSR	5′-GATTACGCCAAGCTTACCCTGCCTGGCTCGTGGGCGCGTAT-3′
For genome walking	
PyPSY-GR	5′-GGCAAGGGAGCACCTGCAAACGCCAAGG-3′
PyPSY-NGR	5′-ACAGCATCCATTGGCGGCGGATTGAGGCT-3′
For plasmid construction	
PyPSY-pF	5′-GAAGGATTTCACATATGATGAGCCTCAATCCGCCG-3′
PyPSY-pR	5′-GTTTTATTTGAAGCTTCTACCCAGAACCAAACGACAC-3′
For colony PCR	
PyPSY-CF	5′-AGGCGTTTGTGTCGTTTGG-3′
PyPSY-CR	5′-TACTCAGGAGAGCGTTCACC-3′

## Data Availability

The sequence data were deposited in NCBI under the accession number PP789597.
